# The impact of an operation and management intervention on toilet usability in schools in the Philippines: a cluster randomised controlled trial

**DOI:** 10.1186/s12889-019-7833-7

**Published:** 2019-12-16

**Authors:** Helen Buxton, Jed Dimaisip-Nabuab, Denise Duijster, Bella Monse, Habib Benzian, Robert Dreibelbis

**Affiliations:** 1 Disease Control Department, London School of Hygiene and Tropical Medicine, Kepple Street, London, UK; 2 Department of Epidemiology and Biostatistics, College of Public Health, University of the Philippines, 625 Pedro Gil St, Ermita, 1000 Manila, Metro Manila Philippines; 3 Department of Social Dentistry, Academic Centre for Dentistry Amsterdam, University of Amsterdam and VU University, Gustav Mahlerlaan 3004, 1081LA Amsterdam, The Netherlands; 4 Regional Fit for School Programme, Gesellschaft für Internationale Zusammenarbeit (GIZ), L.P. Leviste corner Rufino Street, Makati City, Metro Manila Philippines; 5 Department of Epidemiology and Health Promotion, WHO Collaborating Center for Quality Improvement and Evidence-based Dentistry, College of Dentistry & College of Global Public Health, New York University, 433 First Avenue, New York, NY 10010 USA

**Keywords:** School sanitation, Operation and management, Joint monitoring programme

## Abstract

**Background:**

Access to usable water, sanitation and hygiene provision in schools is included within indicators in the Sustainable Development Goals. Progress towards these indicators is dependent on developing an understanding of which intervention components are most effective to operate and maintain usable services. This study aimed to determine the impact of a school toilet operation and management intervention in the Philippines on toilet usability and student and teacher satisfaction, adjusted for clustering at school level.

**Methods:**

In a non-blinded cluster randomised controlled trial, we compared improvements in usability and cleanliness of school toilets among those schools receiving a low-cost, replicable intervention. Toilet usability was measured based on Sustainable Development Goal indicators related to school sanitation defined by the UNICEF/WHO Joint Monitoring Programme for Water, Sanitation and Hygiene. Intervention schools received consumables, support kits, and structured tools designed to facilitate operation and maintenance of sanitation facilities. The primary outcome, toilet usability and cleanliness, was compared through a difference-in-difference analysis of toilet usability. Secondary outcomes of student and teacher satisfaction were measured through a survey at endline. All outcomes were adjusted for clustering at school level.

**Results:**

20 eligible schools in the Batangas region of the Philippines were randomly selected and allocated to either control or intervention group. We found that non-classroom toilets were 48% more likely to meet quality benchmarks in intervention schools, but this was not statistically significant. When including in-classroom toilets in the analysis, there were no significant differences in toilet usability - defined as accessible, functional, private and of high quality – between intervention and control schools. When stratified by toilet location, children in the intervention group clusters expressed a minor, but statistically significant increase in overall satisfaction with sanitation facilities (*p* = 0.035).

**Conclusion:**

Water, sanitation and hygiene interventions in schools focusing on operation and maintenance showed potential to improve toilet usability, but universal achievement of SDG targets may require additional efforts addressing toilet infrastructure.

**Trial registration:**

ClinicalTrials.gov NCT03204175, June 2017 prior to participant enrolment.

## Background

The provision of adequate water, sanitation and hygiene (WASH) facilities and services in schools (WinS) and improvements in WASH-related behaviours among school-aged children are associated with a range of education and health benefits, including reduction in absenteeism [1], increased enrolment of girls [2], and reduction in respiratory infection [3]. However, these benefits cannot be realised if facilities are not functional, or if necessary WASH infrastructure remains locked and out of use due to lack of resources to manage operation and maintenance (O&M) [4, 5]. Inadequate or poorly maintained facilities may also be an active deterrent to toilet use and/ or pupil handwashing practice [6].

The Sustainable Development Goals (SDGs) include multiple WinS targets and indicators. The Joint Monitoring Programme for Water Supply, Sanitation and Hygiene of UNICEF and WHO (JMP) has developed service ‘ladders’ for WinS, which enable progress to be tracked against steps on the ladder classified as no service, limited service, basic services and advanced services. Basic school sanitation is defined as improved facilities which are sex segregated and usable. ‘Usable’ is defined as accessible (doors are unlocked or a key is available at all times), functional (toilet is not broken, toilet hole is not blocked and water is available for flush/ pour flush toilets), and private (closable doors that lock from the inside, and no large gaps in the structure) [7]. The JMP indicators record achievement as a binary indicator of usable facilities at school-level and are not designed to monitor variances in quality of facilities within each school. Without further disaggregation of “usability” into its subsequent measures of accessibility, functionality and privacy, it is difficult for regional or national policy to address existing gaps in WinS coverage. Expanded questions within the JMP enable measurement of indicators of toilet acceptability, accessibility, availability and quality, which may be more relevant for monitoring service provision in middle income countries, yet JMP tools do not provide insight on teacher and student’s drivers of use of WinS facilities [8].

Intervention studies have identified key components required for effective school sanitation management beyond the provision of infrastructure. These include budget allocation; student and parent monitoring [9]; provision of small-scale infrastructure such as handwashing facilities (HWFs); provision of consumables (soap and cleaning supplies); school health clubs [10] and the use of local champions to promote WASH [11]. Progress towards WinS indicators is facilitated by developing a better understanding of how best to leverage the additive benefits of these components.

The Fit for School approach (FIT) is developed to integrate evidence-based interventions for improving child health outcomes into broader government-led school-based programmes by focusing on simple, scalable, sustainable and system-oriented interventions. Interventions include the construction of group HWFs, daily group handwashing with soap and daily group toothbrushing with fluoride toothpaste. With technical support from Deutsche Gesellschaft für Internationale Zusammenarbeit (GIZ) Fit for School Program, the Department of Education in the Philippines (DepEd) has been integrating the FIT approach into its school health programming since 2009. Previous evaluation studies of the FIT approach have found significant reduction in the presence of dental caries, and mixed results of impact on prevalence of moderate to heavy soil transmitted helminth infections and body mass index [12, 13]. Programme evaluation points to challenges with provision of budget for consumables such as soap for handwashing; and capacity of school staff to clean and maintain school toilets [14]. In response to these challenges, the FIT approach was extended to include a school sanitation operation and maintenance package (FIT Plus). The extended approach is designed to complement the DepEd WinS policy [15] which stipulates that all schools are responsible for managing usability and cleanliness of sanitation school sanitation facilities and must provide WinS monitoring data.

We conducted a cluster randomised controlled trial to measure the impact of the FIT Plus intervention on toilet usability and student and teacher satisfaction with toilet facilities in 20 schools in the Batangas region of the Philippines. A cluster design was utilised to account for school level O&M procedures. This mixed methods evaluation combined facility assessments, qualitative data collection and analysis, observational data and survey data to assess program adherence and sanitation-outcomes.

## Methods

### Context

The study was conducted in the Batangas Province of the Philippines, a lower-middle-income country [16]. Toilets in study schools are predominantly flush or pour flush, connected to on-site septic systems. Toilets often have either taps or water containers inside for flushing and/or personal hygiene e.g. anal cleansing purposes. Toilets are either located in classrooms or within school premises. Classroom toilets are not sex segregated and are located in a single classroom with a sink immediately outside the toilet entrance. These toilets are managed by the classroom teacher. Non-classroom toilets are typically sex segregated and organised into blocks with multiple toilet facilities within a single room, separated by dividers. During preliminary consultations, teachers reported that students washed and/or rinsed hands at sinks and basins with taps as well as with buckets with taps or dippers located inside of toilets that are also used for anal cleansing and flushing. HWF were defined as any place ‘it is possible to wash and rinse hands’ and included sinks, basins with a tap, or a bucket in the toilet facility with either a dipper for pouring water or a tap.

Block toilets often have multiple HWF with tap and sink inside the room. Individual toilets are within the school building or a separate structure with HWF (located inside the toilet or right outside the individual cubicles). In both control and intervention groups the responsibility for ensuring toilets were kept clean was assigned to specific teachers, but cleaning duties were often shared with students and parents. Some schools have a janitor assigned to clean non-classroom toilets. These janitors are also in-charge of other school errands and receive minimal salary through parent contributions.

### Study design

This was a cluster randomised controlled trial of a toilet O&M intervention involving 20 schools. The intervention was delivered over a four-month period (August – November 2017) and a difference in difference analysis was conducted to compare toilet usability and toilet quality as well as student and teacher satisfaction levels, clustered by school at baseline and endline.

### Sample selection and allocation

Our sample was restricted to 20 schools due to resource availability. Out of a potential 690 primary schools in Batangas, 39 primary schools were identified by DepEd as meeting the inclusion criteria for this study: school population of 200–999 students; accessible location (within 2 hours from Batangas city centre with mobile phone signal); secure with stable terrain for vehicle access; access to water source; at least one in-use toilet facility and HWF; and at least one multi-story building. Inclusion of multi-story buildings within the selection criteria was to ensure all study schools had a mix of classroom toilets; usually located in single story buildings, and block toilets; typically located in multi-story buildings. Our selection criteria ensured that we selected a sample that was both feasible and accessible for the study that would also reflect the greatest variability in school sanitation infrastructure. Preliminary visits by the GIZ study team excluded ten schools. The research coordinator generated a random number between 0 and 1 in MS Excel for each of the remaining 29 schools and selected the 20 schools with the lowest number to be allocated to either the control arm or to receive the intervention based on order of ascension. Consent was sought and gained from the Principal at each of the 20 selected schools, and parental support was sought from the parents of all children grade 4 and above to participate in the satisfaction survey. We assessed balance by conducting a t-test between intervention and control groups based on school population, and number of toilets by type per school. There were no significant differences found between the groups (Table 1). All toilets in each school which were intended for use by children were included in each school cluster and 16 children grades 4 and above per school were randomly sampled on the day of data collection from the list of children whose parents had given prior consent to participate in the satisfaction survey.

**Table 1:** Toilet Usability Index (TUX) questions and response options mapped against SDG core questions on school sanitation

Attribute	TUX Questions^a^	Response	SDG core question code^b^
Accessible: children can access the toilet whenever they need to	Is the door locked to students?	y/n	S2
	*Is this toilet accessible to intended users?*	y/n	S2
	*Is the key available to children at all times?*	y/n	S2
Functional: children can flush the toilet	How is water supplied to this toilet?	No water available/ bucket and dipper/ piped water	S2
	*Is the toilet bowl broken?*	y/n	S2
	*Is the toilet hole blocked*?	y/n	S2
	*Are the walls and roof stable/ no large cracks*	y/n	S2
Private: children can use the toilet without concern of being walked in on or seen	Is it possible to see inside the toilet compartment from outside (e.g. large cracks or holes in wall)	y/n	S2
	Does the toilet cubicle have a door or a curtain?	no/ curtain only/ door	S2
	Does the door close completely	y/n	S2
	Does the door lock from inside?	y/n	S2
High Quality:	Is there excess mud inside the toilet cubicle?	A lot/ some/ none	XS5
	Is there any litter inside the toilet cubicle?	A lot/ some/ none	XS5
	Are there any traces of faeces in the toilet bowl?	A lot/ some/ none	XS5
	Is there a strong or unbearable odour inside or outside the toilet?	A lot/ some/ none	XS5
	Are there visible traces of faeces inside the cubicle (including the walls, floors and slab)?	A lot/ some/ none	XS5
	Is there a puddle of stagnant water or urine inside the cubicle?	A lot/ some/ none	XS5
	How many flies are there inside the cubicle?	A lot/ some/ none	XS5
	Is there adequate lighting inside the cubicle?	A lot/ some/ none	XS11
	*Is there means for girls to wash themselves inside the toilet cubicle?*	y/n/ not applicable (boy’s toilet)	XS1
	*Are there garbage disposal bins available?*	Yes, with a lid/ Yes, but no lid/ No	XS1
	*Is there adequate ventilation in the toilet cubicle?*	y/n	XS5
	*Is there anal cleansing material available?* *NOTE: Only say yes if there is: EITHER [water and a dipper] OR[a hose] OR [a tap] OR [toilet paper]*	y/n	XS10

^a^Items in italics were not included in final analysis (see Methods for details)

^b^Code corresponds to SDG core questions for monitoring WinS [8]

### Intervention description

The intervention was designed based on a theory of change (TOC) which is articulated in Additional file 1. The intervention was designed through consultation and piloting with one large school in Manila. The intervention enabled schools to implement daily group hand-washing and tooth-brushing activities and to proactively manage school sanitation facilities and ran from August 2017 – November 2017. An orientation day for intervention school management teams was held at baseline and a member of staff at each school was identified to champion the intervention. Intervention schools received a detailed manual on toilet O&M that included WinS monitoring worksheets, budget allocation exercises, example cleaning rotas and checklists for schools to use. Content for the manual was developed based on DepEd requirements and feedback from the consultation school. The O&M manual is in English and targets teachers and school principals. In addition, an O&M orientation video in both English and Tagalog was developed to orient parents and school communities. Completion of the WinS monitoring tools is positioned as the initial step in the ToC as it enabled schools to allocate adequate resources to sanitation management. The tools enabled schools to systematise O&M in the way that was most appropriate to each school. Intervention schools also received basic infrastructure to set up group HWFs; a toilet user’s kit (including toilet brush, trash can, bucket and dipper); cleaning tools for each toilet; a basic maintenance kit for each school; and a monthly supply of hygiene consumables such as cleaning supplies, soap and toothpaste. The school Principal, assisted by the WinS champion, are responsible for filling in the plan and resource mapping templates. The Principal ensures that cleaning checklists are completed as an indicator that regular cleaning is being done. A member of the intervention team visited schools once each month to deliver consumables and provide guidance if any issues were identified. The sanitation-specific outcomes of the FIT Plus approach were hypothesised to be more usable toilets available at each school and higher levels of satisfaction with school toilets experienced by both children and school staff. Schools in the control group received the intervention components at the end of the study period.

### Primary and secondary outcome measures

The primary outcome measure was the usability of school sanitation, and the secondary outcome was student and school staff satisfaction with toilet provision. The primary outcome was assessed via the toilet usability index (TUX); and the secondary outcome was measured by a satisfaction survey. Results from both tools were adjusted for clustering at school level during analysis. The TUX tool was developed and tested on hand-held digital devices in April 2017 in 16 schools in the Philippines which were not part of the study, following the same criteria as the study to select schools for inclusion. The TUX was developed in English, and all enumerators spoke adequate English to use and test the tool. TUX baseline data was collected in early August 2017, 2 weeks prior to the implementation of the intervention. The TUX and the satisfaction survey of students and teachers were conducted at endline in November 2017.

### Toilet usability index (TUX)

The TUX was designed to mirror global SDG indicators for school sanitation [8] using the toilet as the primary unit of analysis. In addition to the three criteria stipulated in the JMP definition of usability: accessibility, functionality and privacy, the TUX includes consideration of the JMP expanded questions concerned with acceptability, accessibility, availability and quality of sanitation provision. The TUX groups these indicators under the umbrella term of ‘quality’ in order to consider a fourth dimension of usability. Following principles of the C-OAR-SE methodology [17], TUX questions were mapped against SDG indicators and refined to maximise content validity. The tool was tested to ensure internal consistency and inter-rater reliability. The definitions of toilet usability attributes and the final set of questions of the TUX are presented in Table 2. The TUX data was collected using pre-programmed handheld digital devices running Open Data Kit software [18].

**Table 2:** School characteristics and assessment of balance between control and intervention groups

	Control	Intervention	*P* value
Average number of children enrolled per school	450	425	0.69
Average number of multi-story buildings per school	2	2.4	0.33
Average number of classroom toilets per school	6	6.7	0.69
Average number of non-classroom toilets per school	2.7	2.5	0.76

To measure usability, we assessed if the toilet was accessible, functional, private and of high quality through a combination of observable characteristics assessed through direct observation by the toilet by study staff. For non-classroom toilets organized into blocks, data were collected for each toilet independently as well as information about the communal space within the block. A number of variables specific to each domain of quality that were part of the original TUX were not included in the final analysis. Accessibility was consolidated as door to the toilet is not locked. Based on observations during data collection, toilets that were locked remained locked at all times and keys were not available for students; all aspects of accessibility were captured with the single question. Functionality was reduced to an indicator of water being available for flushing. Among study schools, water was either not provided or turned off in toilets where the bowl was broken, pipes blocked, or if the walls and roof were not stable; therefore, water availability was better proxy measure for functionality than other developed indicators. Our privacy measure retained all four JMP requirements: i) presence of a door/curtain; ii) that closes completely; iii) can be locked from inside and iv) has no large gaps/ holes in the structure. JMP requirements for toilets to be lockable from the inside do not apply to pre-primary children, and data from toilets for use by kindergarten children were adjusted accordingly. We included 12 quality indicators in data collection (Table 2). Responses were collected on a three-point scale (a lot, some, none), with the exception of binary questions on means for girls to wash themselves, garbage bins, and facilities for anal cleansing (e.g. water and a dipper; a tap; a hose; or toilet paper). We observed a bimodal distribution among all observed variables and simplified all measures to binary presence / absence indicators. In order to combine our quality measures into a single indicator, we assessed the internal consistency (Cronbach’s alpha) of observed variables in both control and intervention schools and removed indicators one by one until we had maximized internal consistency. Our analysis identified 8 of the quality indicators which gave consistently similar responses: odour; faeces on walls/floor; flies; lighting; urine or stagnant water on floor; mud on the floor; faeces in the bowl; and visible litter. For ease of analysis, the quality score was scaled to be out of 10, by dividing the score by the number of indicators (8) and multiplying by 10. For non-classroom toilets, scores for individual toilets and the block in which they were located were averaged. For the purposes of our analysis, a rating of high quality was assigned to toilets which reached scores of 8.5/10. Analyses of each indicator occurred in sequence: functionality was assessed only among toilets that were accessible; and privacy and quality only assessed among toilets that were functional.

### Satisfaction survey

Student and teacher satisfaction with school toilets was assessed through two surveys conducted at endline developed based on formative qualitative research. Qualitative research was conducted 2 weeks prior to the implementation of the intervention, to explore factors that determine student and teacher satisfaction with toilet facilities. Details of the qualitative study are described (See Additional file 2). Qualitative data was mapped against the JMP criteria for toilet usability [8], to develop questions which were internally consistent with children’s and teachers’ own perception of these criteria. For the children’s survey response choices were designed to minimize ambiguity, interactive graphics were used to capture data, and all questions were administered in local language [19]. Sixteen children from grade 4 and above were randomly selected to be surveyed from each school (320 in total). Four teachers, appointed to participate by the Principal, were surveyed in each school (80 in total). The average score per indicator of satisfaction was calculated and results compared across control and intervention groups.

### Intervention Fidelity

Fidelity of the intervention (the extent to which intervention supplies were delivered to schools as planned) and compliance (the extent to which schools followed recommended procedures and implemented activities) were measured through interviews with school Principals and additional questions were also integrated in the children’s survey to measure compliance.

### Analytic methods

Similar analytic approaches were used for all primary and secondary outcomes. For binary outcome measures (toilet usability and process indicators), we calculated a risk ratio (RR), which provides a measure of the difference in probability between intervention and control. Risk ratios were approximated through the use of generalised estimating equations with the log link -Poisson distribution and robust standard error terms, which enabled adjustment for clustering. For continuous outcome measures (satisfaction), we assessed the mean difference between intervention and control groups using generalised estimating equations with normal distribution.

When measures were available at both baseline and endline, difference in difference analysis approaches were used, which provide risk ratio or a mean difference between toilets in the intervention group compared to the control group, after adjusting for differences at baseline. If baseline data were not available, only endline data were used. All analyses were adjusted for school-level clustering.

## Results

### Sample characteristics

The average number of children per school was 449 in control schools and 420 in intervention schools. Control schools had a slightly higher average number of toilets than intervention schools (13 vs 11) and intervention schools had a higher proportion of block toilets than classroom toilets (59% in intervention group vs 50% in control group). Over 80% of toilets, including all classroom toilets, in both control and intervention schools were not segregated by gender. As a result we excluded consideration of gender segregation from our measurement of toilet usability.

### Intervention fidelity and compliance

All 10 intervention schools received the intended intervention, and 10 intervention and 10 control schools were analysed for the primary outcome. In both intervention and control groups only 3 schools had completed the DepEd WinS monitoring processes by endline data collection. This was positioned as the preliminary step in the FIT theory of change, which would enable each school to develop a work plan for sanitation improvements. Other process outcomes are reported in Table 3. Overall there was an increase in the number of intervention schools reporting increased parental involvement in toilet O&M and the use of cleaning schedules. Data from monthly visits by the implementation co-ordinator indicate varying degrees of compliance (Table 3), but all schools had erected the HWF at schools by the end of the intervention period, indicating engagement with the programme.

**Table 3:** Intervention fidelity and compliance (as reported by school Principal) by intervention and control status

	Control	Intervention^a^
Schools which completed WinS monitoring	3/10	3/9
School which received the toilet maintenance kit	n/a	9/9
Schools with toilet cleaning schedule	5/10	8/9
Schools where parents play active role in toilet O&M	6/10	8/9
Schools which had assembled HWF within one month^b^	n/a	8/10
Schools which had assembled HWF within two months^b^	n/a	9/10
Schools which had assembled HWF within three months^b^	n/a	10/10

^a^Process data missing for one intervention school

^b^verified through monthly monitorring

### Impact on toilet usability and quality

In the control group we identified a total of 133 toilets at both baseline and endline compared to 113 toilets at baseline and 111 at endline in the intervention group. Coefficients for intra-cluster correlation (ICC) values at baseline were higher than anticipated for all variables of interest. At endline we found no significant difference in the percentage of toilets that were accessible comparing the 10 control and 10 intervention schools, when differences at baseline were controlled for (RR: 0.9, *p*-value: 0.737) (Table 4).

**Table 4:** Toilet usability and quality at baseline and endline among intervention and control schools

	n	Baseline	n	Endline	Difference	DiD Risk Ratio (95% CI)	*p*-value
Accessible							
Control	131	84%	132	83%	-1%	0.92	*p* = 0.737
Intervention	124	85%	124	77%	-8%	(0.63 – 1.16)	
ICC		0.211					
Functional^a^							
Control	110	95%	109	88%	-7%	0.94	*p* = 0.271
Intervention	105	90%	95	89%	-1%	(0.85 – 1.05)	
ICC		0.349					
Private^b^							
Control	104	54%	95	54%	0%	0.80	*p* = 0.280
Intervention	96	54%	85	44%	-5%	(0.50 – 1.22)	
ICC		0.211					
High quality^b^							
Control	104	69%	96	64%	-5%	1.07	*p* = 0.660
Intervention	95	68%	85	73%	5%	(0.70 – 1.45)	
		0.329					
Usable^c^							
Control	131	30%	132	24%	-6%	0.86	*p* = 0.637
Intervention	124	29%	124	25%	-4%	(0.50 – 1.63)	
ICC		0.149					

^a^Functional was only assessed among toilets that were accessible

^b^Privacy and quality only assessed among toilets that were both accessible and functional

^c^Usable defined as toilets that were accessible, functional, private and of high quality

Reasons given by the Principal for inaccessible toilets were: septic tank issues (5%), conversion into other purposes (e.g. storeroom) (9%), damaged toilets (6%), assigned for demolition (17%), or kept locked due to limited capacity to manage cleaning (4%).

We found no significant difference between control and intervention groups for changes in functionality, privacy or overall usability (Table 4). Disaggregated privacy indicators reveal that most toilets were classified as non-private because they do not lock from the inside.

We stratified our analysis by classroom and non-classroom toilets in order to account for differences in accessibility, visibility, and management of these facilities (see Additional file 3). Non-classroom toilets in the intervention group had a 48% higher likelihood of being classified as high quality compared to non-classroom toilets in the control group, though this was not statistically significant (RR: 1.48, *p*-value: 0.118). We also observed a 22% decrease in the probability that a classroom toilet would be classified as high quality in intervention schools compared to control schools, also not statistically significant (RR: 0.78 *p* = 0.147).

To account for differences in school size, data were translated into ratio of the number of students to toilets that met each usability indicator. No significant differences were observed between control and intervention groups for any of the criteria of toilet usability (See Table 5).

**Table 5:** Student to toilet ratio at baseline and endline by usability and quality measures among intervention and control schools

	Accessible toilets^a^		Functional toilets^b^		Private toilets^c^		High quality toilets^d^		Accessible, Functional, Private and high quality ^c^	
	Baseline	Endline	Baseline	Endline	Baseline	Endline	Baseline	Endline	Baseline	Endline
Control	44	46	40	41	87	90	48	63	119	149
Intervention	45	48	44	43	80	136	53	48	145	208
*Mann - Whitney*	*p = 0.28*		*p = 0.283*		*p = 1.000*		*p = 0.65*		*p = 0.471*	

^a^Accessibility defined as door is not locked

^b^Functional defined as water is available for flushing in either cubicle or block

^c^Privacy defined as: no large gaps /hole in structure, toilet has a door, which closes completely, and locks from the inside. Toilets intended for pre-primary children can be classified as private without a lock on the inside

^d^High quality toilets defined as those which scored more than 8.5/ 10 across a range of quality indicators

### Impact on children and teacher satisfaction with toilet facilities

Overall children in the intervention group expressed no significant difference in perception of accessibility or privacy of toilets from children in the control group, however children in intervention schools were 30% more likely to consider their toilets functional (RR 1.30, *p* value = 0.005) (Table 6). Satisfaction scores among children in the intervention group were an average 0.4 points higher than children in the control group, and this difference was significant (*p* = 0.035). Analysis of disaggregated quality indicators (Additional file 4) suggests that children in the intervention group were more likely to report finding the toilet flushed at last use, that there was no urine on the floor, and that toilets did not smell.

**Table 6:** Children’s satisfaction with toilet facilities at endline among student attending in intervention and control schools

	Control *n* = 160	Intervention *n* = 160	RR or Mean Diff (95% CI) *P*-value
Accessibility: Children say they can access the toilet whenever they need to	63%	69%	1.1 (0.77–1.54) *p* = 0.637
Functionality: Children say their toilet has everything they need	61%	80%	1.3 (1.09–1.567) *p* = 0.005
Privacy: Children say they do not worry about people walking in on them when they are using the toilet?	52%	48%	0.9 (0.75–1.18) *p* = 0.596
Quality scoring (10-point score)	8.62 (1.62)	9.05 (1.31)	+ 0.43 (0.03–0.84) p = 0.035

Teachers’ reported increased satisfaction with provision of materials for cleaning (RR: 2.3, *p* = 0.002) and provision of materials for use e.g. soap for washing hands (RR: 2.0, *p* = < 0.001) (Table 7). However, there was no significant difference in teachers’ level of satisfaction with toilet cleanliness or children’s behaviour when using toilets between control and intervention groups.

**Table 7:** Teachers’ satisfaction with toilet facilities at endline among teachers at intervention and control schools

Percent of respondents satisfied with …	Control	Intervention	RR (CI)	(*p*-value)
Conditions of children’s toilets	37%	45%	1.2 (0.67–2.09)	*p* = 0.522
Cleanliness of children’s toilets	32%	42%	1.3 (0.66–2.59)	*p* = 0.442
Responsibilities for how cleaning is assigned	45%	55%	1.2 (0.72–2.07)	*p* = 0.453
Materials available for cleaning toilets	23%	75%	2.3 (1.36–3.91)	*p* = 0.002
Children’s behaviour when using toilets	20%	30%	1.5 (0.42–5.42)	*p* = 0.536
Availability of toilets for children	82%	97%	1.2 (1.04–1.35)	*p* = 0.014
Possibility for children to flush toilets	37%	70%	1.9 (1.12–3.10)	*p* = 0.016
Availability of materials for anal cleansing	42%	87%	2.1 (1.35–3.14)	*p* = 0.001
Availability of materials for HWWS	45%	90%	2.0 (1.35–2.95)	*p* < 0.001
Privacy of children’s toilets	67%	87%	1.3 (1.06–1.59)	*p* = 0.013

## Discussion

This trial was designed to assess the impact of a scalable, low-cost, school toilet O&M and hygiene promotion intervention on toilet conditions and children’s satisfaction with school toilet facilities. We hypothesised that the intervention would increase the number of usable toilets and the quality of usable toilets. We found that non-classroom toilets in the intervention group had a higher likelihood of being classified as high quality than non-classroom toilets in the control group, though this was not statistically significant. When including classroom toilets in the analysis, we noted no difference in the proportion of usable toilets in schools that received the FIT Plus intervention at endline compared to those that did not.

There are a number of possible explanations. First, higher than anticipated ICC values at baseline made it difficult to detect statistically significant effects. However, we do still demonstrate a large change in quality of non-classroom toilets and the ICC values support an interpretation that this change was meaningful even if not significant. In addition, baseline rates of usability indicators were high - leaving little room for improvement. It is common practice in the Philippines for parents and teachers to spend a week readying schools and facilities for the coming school year following the holidays, and this may explain relatively good conditions observed at baseline, which was conducted directly after the holidays. It was not possible to blind schools to the study arm they were assigned to as intervention schools were provided with hardware and consumables and requested to conduct group hygiene activities. However, as results are similar in both control and intervention groups we do not think that knowledge of study arm impacted on how the schools reacted to the intervention. Nonetheless, principals, school staff and children were highly reactive to the presence of the data collection team. In all schools, Principals engaged children and teachers in active toilet cleaning when data collection teams arrived. We minimised this by explaining to school staff we wanted to see the real conditions and by standardising the time of data collection; however, results reflect to some extent recent cleaning in response to the presence of the study team. By endline, schools were more accustomed to visitors and reactivity greatly reduced in both study groups. Our follow-up period was quite short - we were only able to assess changes in usability over a three-month period (mid-August – mid November). Given high rates of reactivity of baseline and the gradual attenuation in reactivity, it is plausible that larger differences between control and intervention schools would have been seen at later time points. An area for future research may be to revisit these schools at a later date and see is the results were maintained.

Process data reveals schools had some challenges complying with intervention requirements, which may have contributed to the limited effect of the intervention. The TOC assumed that schools could be enabled to better manage their sanitation facilities through the provision of structured tools and worksheets, yet only three schools in the intervention group had completed the WinS monitoring tool by the end of the intervention period. This tool was positioned as the precursor to intervention implementation and represents a significant gap in compliance. Achievement of the other critical steps outlined in the TOC was reached by a higher number of intervention schools than control – but no step was achieved by all intervention schools. However, more intervention schools than control schools reported use of cleaning schedules and parental involvement in the steps intended to assist schools to better manage facilities. The intervention design relied heavily on the role of a ‘champion’ at each school to manage distribution of supplies and encourage uptake of O&M tools by teachers. Future interventions may need to do more to activate this role. Management tools provided to schools were designed so that teachers could create systems that worked for their specific context, and the division of responsibilities (between parents, teachers or children) was determined by each school. All intervention schools had cleaning schedules in place compared to only half of control schools yet this did not result in significant changes in results. A potential focus of future research may be to look at which actors are best placed to hold responsibility for regular cleaning. Overall the intervention relied on uptake of the management tools supplied in the manual, but as acceptability and utility of these tools was piloted in a large urban school, future interventions could benefit from further participant-led development of these tools and required training to use them. Challenges with compliance are common to WinS interventions [1, 4, 11, 20]. Randomized trials of school-based interventions in Kenya and Mali found a relationship between school-level compliance with WASH interventions and better pupil level outcomes [21, 22]. How to provide schools with the appropriate oversight and support to manage school WASH interventions requires further investigation.

Our TOC assumed that a major barrier to accessibility was the school’s capacity to manage facilities and that schools deliberately limited accessibility to some facilities in order to reduce the effort needed to maintain all school latrines. Yet at baseline school Principals only identified a small proportion of toilets per school which were kept closed due to incapacity to keep them clean, and a larger proportion of toilets were closed due to septic tank issues or damage. Hence, to see a larger increase in toilet usability among study schools would have required additional structural repairs to toilets, septic tanks, basins, water supply, walls, and doors. Although schools were provided with a basic maintenance kit, large-scale structural repairs or improvements were beyond the scope of the intervention. The lack of improvement in structural conditions of school toilets is consistent with other evaluations of WinS programmes that included a sanitation maintenance component [10, 23]. For example, in a randomized trial in Kenya [10], schools were able improve the cleanliness of school latrines after receiving various combinations of financial and organizational support; however, intervention packages were not sufficient to enable schools themselves to improve the structural quality of school latrines. The FIT Plus intervention proposed to solve issues of structural functionality by providing schools with simple hardware tools and strengthening parent-teacher associations to identify funds and budget for repairs [11]. Busy schools may not have had the time to effectively utilize these materials without additional external support or may have been unsuccessful in marshalling community support, a factor that has been associated with improved school sanitation quality [11]. These barriers are not unique to the schools in this study, yet many WinS policies and interventions promote similar approaches. Our study highlights that while interventions focusing on operation and maintenance have the potential to increase toilet quality; this potential is limited unless interventions are also designed to strengthen toilet infrastructure.

Discrepancies between globally defined indicators and end user preferences may also hinder efforts to improve toilet usability in schools. In both study groups overall usability is skewed by low rates of privacy. According to JMP monitoring frameworks [8], a toilet is only classified as private if it locks from the inside. Yet teachers testified to having disabled locks to prevent children from getting stuck inside, and a major concern voiced by children was getting locked inside a toilet. The JMP framework also stipulates that sanitation should be gender segregated, however in the context of our study, classroom toilets intended for use by all genders, had higher rates of usability than gender-segregated toilets. This is assumed to be a result of the clear responsibility of the classroom teacher including constant supervision when the toilet is located within the classroom. The JMP framework aims to provide universally measurable indicators, a necessary component for tracking global progress to meeting Sustainable Development Goal Targets. Our findings highlight the potential to lose some context specific detail in such an approach, and future participant-led approaches can help further develop and refine locally appropriate indicators to complement global monitoring data.

User satisfaction with facilities provides subjective validation of our measure of toilet usability. For the majority of variables measured in the children’s satisfaction survey there was no significant increase among children in intervention schools, findings which mirror those indicated by the TUX tool. Children in the intervention group were more likely to state that their toilet has everything they need, and follow-on questions isolated soap as the component which was more likely to be found in intervention than control groups. This indicates both that the intervention was successful in increasing children’s access to soap, and that soap has been internalised by the children as something which they ‘need’. Teacher surveys also found a significant increase in teachers’ satisfaction levels with availability of cleaning consumables – suggested that this component is desirable to teachers.

We note a number of limitations to our study. Although the inclusion criteria were quite restrictive, our aim was both to ensure accessibility would not impede study delivery, and to increase the applicability of study results by ensuring there was variation in toilet provision within the sample. However, we recognise that the criteria used may affect the generalisability of the results. Even with multiple toilets within the school, our study had a limited sample size and low variability within schools; limiting the ability of our study to detect a significant effect. The limited variability in sanitation quality also constrained our ability to develop a replicable and validated measure of usability. While many of the core and additional JMP indicators of school sanitation quality were not applicable in our study context, we have included these variables in Table 2 for use in future studies. Given the nature of the intervention, neither study participants nor data collection staff were blinded to intervention status. The lack of blinding may have contributed to the high rates of reactivity in our study; however, the lack of a large and consistent impact of the intervention on toilet usability suggests that the effect of non-blinding was likely limited. Our measurement of satisfaction is susceptible to social desirability bias, especially in this context where people have been given things and were then asked how satisfied they are.

## Conclusion

We found an increase in quality in non-classroom intervention toilets when excluding the well-maintained classroom toilets from the analysis, however this was not statistically significant when clustered by school. Detecting intervention effects on toilet usability was hindered by generally high-quality conditions at baseline and limited variability within schools across both control and intervention groups especially within the subset of classroom toilets. Achieving usability of all toilets may require increased focus on capital investment and hardware improvements, alongside tools to improve operation and maintenance.

## Supplementary information

**Additional file 1.** Theory of Change of the FIT Plus approach.

**Additional file 2.** Formative qualitative research methods.

**Additional file 3.** Toilet usability stratified by toilet type.

**Additional file 4.** Intervention impact on children’s satisfaction.

## Data Availability

The datasets generated during and/or analysed during the current study are available from the corresponding author on reasonable request.

## Abbreviations

DepEd: Department of Education in the Philippines
FIT: Fit for School
GIZ: Deutsche Gesellschaft für Internationale Zusammenarbeit
HWF: Handwashing facility
JMP: The Joint Monitoring Programme for Water Supply, Sanitation and Hygiene of UNICEF and WHO
O&M: Operation and Management
RR: Risk ratio
SDGs: Sustainable Development Goals
TOC: Theory of change
TUX: Toilet usability index
UNICEF: United Nations International Children’s Emergency Fund
WASH: Water, sanitation and hygiene
WHO: World Health Organisation
WinS: WASH in Schools
