# Supplementary material for: The impact of an operation and management intervention on toilet usability in schools in the Philippines: a cluster randomised controlled trial
Source: BMC Public Health. 2019 Dec 16;19:1680. doi: 10.1186/s12889-019-7833-7 (PMC6916048; doi:10.1186/s12889-019-7833-7)
Supplement: Supplementary file 1 — Additional file 1. Theory of Change of the FIT Plus approach. [file 12889_2019_7833_MOESM1_ESM.docx]

Additional file 1: Theory of Change of the FIT Plus approach


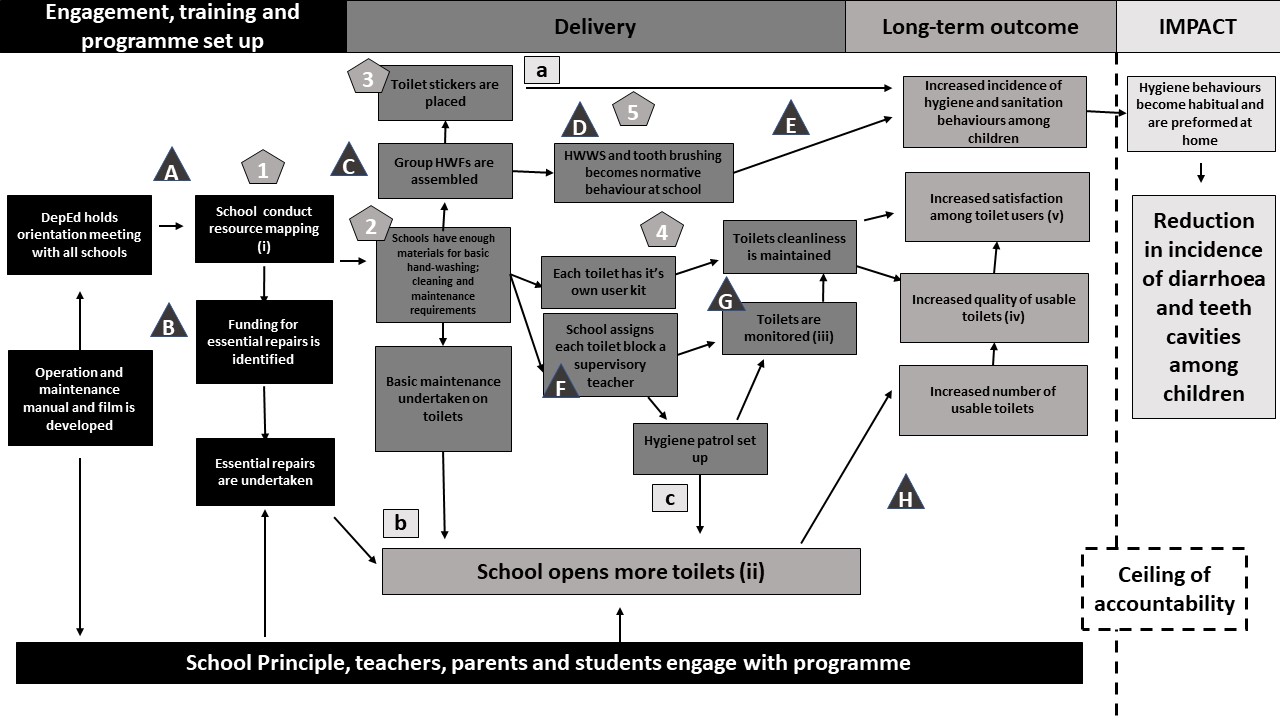


| Assumptions  **A** | Interventions |
| --- | --- |
| 1. Principals are engaged with the programme and have the time to commit to programme 2. There are funds available within the community 3. PTA will be available and willing to assemble 4. Schools will have capacity to run daily group activities 5. Group activities will have an effect on individual behaviour 6. Schools have capacity to run supervisory activities 7. Monitoring will result in cleaning of toilets 8. Assumes DepEd have monitoring system in place at time of intervention | 1. Delivery of O&M manual to assist PTA to conduct resource mapping 2. FIT Plus toilet kits and components for group HWF are delivered 3. Hygiene stickers are placed at hand-washing locations 4. FIT Plus team pay weekly visits to the schools to build capacity and problem solve 5. Daily group HWWS and tooth-brushing at school |
| Rationale | Indicators (i) |
| 1. Evidence from systematic reviews that behaviour can be cued by reminders 2. Observational evidence that some toilets are kept locked due to disrepair 3. Observational evidence that some toilets are kept locked due to inability to keep clean | 1. Resource mapping tool completed for each school within 1 month of start of intervention period 2. Increase in number of toilets open at school as reflected in baseline and end-line figures 3. Toilet monitoring tools completed for each school 4. 50% increase in toilet quality score in intervention vs control arm by end of intervention 5. Satisfaction scores are 20% higher in intervention than control arm. |
