# Supplementary material for: The impact of an operation and management intervention on toilet usability in schools in the Philippines: a cluster randomised controlled trial
Source: BMC Public Health. 2019 Dec 16;19:1680. doi: 10.1186/s12889-019-7833-7 (PMC6916048; doi:10.1186/s12889-019-7833-7)
Supplement: Supplementary file 2 — Additional file 2. Formative qualitative research methods. [file 12889_2019_7833_MOESM2_ESM.docx]

## Additional file 2: Formative qualitative research methods

For the qualitative components of the study, six schools were randomly selected – three each from the control group and the intervention group.

At each of six randomly selected schools 12 children were randomly selected from those whose parents had given prior consent, stratified by gender and age group. In gender specific groups, children lead enumerators around the school according to a large, pre-drawn map to identify toilets they used most often. Children also identified their favourite and least-favourite toilets. Enumerators selected two children from each group for an in-depth interview. Children were interviewed in pairs to reduce anxiety. Interview questions were designed to identify children’s priorities through qualitative application of factorial vignettes [19]. Children were asked to choose which toilet they would prefer based on a range of assigned attributes – e.g. would they choose a toilet which did no lock but had water to flush, or a toilet which had water but was smelly.

Short in-depth interviews were also held with two teachers, the principal and the janitor (if there was one) per school. Interview guides included questions related to participant’s satisfaction with the toilet facilities and if they felt they were suitable to children’s needs. All interviews and discussions were conducted in local language.
