# Supplementary material for: The impact of an operation and management intervention on toilet usability in schools in the Philippines: a cluster randomised controlled trial
Source: BMC Public Health. 2019 Dec 16;19:1680. doi: 10.1186/s12889-019-7833-7 (PMC6916048; doi:10.1186/s12889-019-7833-7)
Supplement: Supplementary file 3 — Additional file 3. Toilet usability stratified by toilet type. [file 12889_2019_7833_MOESM3_ESM.docx]

#### **Additional File 3: Toilet usability stratified by toilet type**

|  |  | TOTAL* | | Accessible toilets** | | Functional toilets*** | | Private toilets**** | | High quality toilets***** | | Usable****** | |
| --- | --- | --- | --- | --- | --- | --- | --- | --- | --- | --- | --- | --- | --- |
|  |  | Baseline | Follow-up | Baseline | Follow-up | Baseline | Follow-up | Baseline | Follow-up | Baseline | Follow-up | Baseline | Follow-up |
| All toilets | | | | | | | | | | | | | |
| Control | 131 | | 132 | 110/131 | 109/132 | 104/110 | 96/109 | 56/104 | 51/95 | 72/104 | 61/96 | 39/131 | 32/132 |
|  |  |  |  | 84% | 83% | 95% | 88% | 54% | 54% | 69% | 64% | 30% | 24% |
| Intervention | 124 | | 124 | 105/124 | 95/124 | 95/105 | 85/95 | 51/96 | 37/85 | 65/95 | 62/85 | 36/124 | 31/124 |
|  |  |  |  | 85% | 77% | 90% | 89% | 53% | 44% | 68% | 73% | 29% | 25% |
|  |  |  |  | RR: 0.927  (CI: 0.627 – 1.160)  p = 0.737 | | RR: 0.942  (CI: 0.845 – 1.048)  p = 0.271 | | RR: 0.779  (CI: 0.496 – 1.224)  p = 0.280 | | RR: 1.07  (CI: 0.789 – 1.451)  p = 0.660 | | RR: 0.856  (CI: 0.449 – 1.632)  p = 0.637 | |
| All classroom toilets | | | | | | | | | | | | | |
| Control | 63 | | 67 | 58/63 | 57/67 | 58/58 | 57/57 | 29/58 | 31/57 | 46/58 | 38/57 | 23/63 | 20/67 |
|  |  |  |  | 92% | 85% | 100% | 100% | 50% | 54% | 79% | 67% | 37% | 30% |
| Intervention | 46 | | 50 | 46/46 | 46/50 | 46/46 | 46/46 | 15/46 | 15/46 | 37/46 | 28/46 | 13/46 | 10/50 |
|  |  |  |  | 100% | 92% | 100% | 100% | 33% | 33% | 80% | 61% | 28% | 20% |
|  |  |  |  | RR: 1.005  (CI: 0.584 – 1.727)  p = 0.987 | | Correlation >1 | | RR: 0.669  (CI: 0.396 – 1.132)  p = 0.134 | | RR:0.784  (CI: 0.553 – 1.092)  p=0.147 | | RR: 0.581  (CI: 0.264 – 1.275)  p = 0.175 | |
| Non-classroom toilets | | | | | | | | | | | | | |
| Control | 68 | | 65 | 52/68 | 52/65 | 46/52 | 39/52 | 27/46 | 20/39 | 26/46 | 23/39 | 16/68 | 12/65 |
|  |  |  |  | 76% | 80% | 88% | 75% | 59% | 51% | 57% | 59% | 24% | 18% |
| Intervention | 78 | | 74 | 59/78 | 49/74 | 49/59 | 39/49 | 36/49 | 22/39 | 28/49 | 34/39 | 23/78 | 21/74 |
|  |  |  |  | 76% | 66% | 83% | 80% | 73% | 56% | 57% | 87% | 29% | 28% |
|  |  |  |  | RR: 0.881  (CI: 0.552 – 1.112)  p= 0.627 | | RR: 0.897  (CI: 0.484 – 1.664)  p = 0.732 | | RR: 0.971  (CI: 0.508 – 1.852)  p = 0.928 | | RR: 1.483  (CI: 0.349 – 0.901)  p = 0.118 | | RR: 1.313  (CI: 0.556 – 3.199)  p = 0.535 | |

1. Toilets assigned for demolition excluded from analysis
2. Accessibility defined as door is not locked
3. Functional defined as water is available for flushing in either cubicle or block
4. Privacy defined as: no large gaps /hole in structure, toilet has a door, which closes completely, and locks from the inside. Toilets intended for pre-primary children can be classified as private without a lock on the inside.
5. High quality toilets defined as those which scored more than 8.5/ 10 across a range of quality indicators.
6. Usable defined as Accessible, Functional, Private and Clean
