# Supplementary material for: The impact of an operation and management intervention on toilet usability in schools in the Philippines: a cluster randomised controlled trial
Source: BMC Public Health. 2019 Dec 16;19:1680. doi: 10.1186/s12889-019-7833-7 (PMC6916048; doi:10.1186/s12889-019-7833-7)
Supplement: Supplementary file 4 — Additional file 4. Intervention impact on children’s satisfaction. [file 12889_2019_7833_MOESM4_ESM.docx]

## Additional File 4: Intervention impact on children’s satisfaction

|  | Control | Intervention | IRR | Confidence interval | P-value |
| --- | --- | --- | --- | --- | --- |
| Children felt happy last time they went to the toilet | 71% | 72% | 1.01 | 0.85 -1.30 | 0.897 |
| Children say they can always go to the toilet when they need to | 63% | 69% | 1.09 | 0.88 – 1.34 | 0.434 |
| Children say they did not have to walk far to the toilet (last time) | 93% | 89% | 0.95 | 0.89 – 1.02 | 0.174 |
| Children say they did not queue for long for the toilet (last time) | 93% | 90% | 0.97 | 0.89 – 1.06 | 0.471 |
| Children say their toilet always has everything they need | 61% | 80% | 1.30 | 1.09 – 1.57 | 0.005 |
| Children say their toilet was blocked (last time) | 10% | 5% | 1.05 | 0.98 – 1.13 | 0.157 |
| Children say there was water to flush (last time) | 94% | 98% | 1.04 | 0.98 – 1.10 | 0.163 |
| Children say there was a bucket and a dipper? (last time) | 98% | 96% | 0.98 | 0.93 – 1.04 | 0.489 |
| Children say there was soap for anal cleansing (last time) | 59% | 89% | 1.52 | 1.18- 1.95 | <0.001 |
| Children say they did not worry about people walking in on them (Last time) | 62% | 58% | 0.94 | 0.75 – 1.18 | 0.596 |
| Children say they did not worry about people peeping on them (last time) | 57% | 61% | 1.07 | 0.90 – 1.28 | 0.440 |
| Children say they did not worry about people hearing what they were doing inside (last time) | 75% | 77% | 1.03 | 0.91 – 1.15 | 0.655 |
| Children feel happy with cleanliness of their toilets | 80% | 85% | 1.06 | 0.92 – 1.22 | 0.443 |
| Children say toilet was flushed (last time) | 84% | 93% | 1.11 | 1.02 – 1.21 | 0.018 |
| Children say there was poo in the bowl (last time) | 4% | 7% | 0.97 | 0.91 – 1.04 | 0.337 |
| Children say there was urine on the floor (last time) | 9% | 2% | 1.07 | 0.99 – 1.16 | 0.074 |
| Children say there were muddy footprints (last time) | 36% | 24% | 1.20 | 1.01 – 1.41 | 0.036 |
| Children say the toilet smelt bad (last time) | 28% | 17% | 1.15 | 1.02 – 1.30 | 0.020 |
| Children said there were flies or cockroaches in the toilet (last time) | 4% | 2% | 1.03 | 0.98 – 1.07 | 0.270 |
| Children said there was enough light to see properly (last time) | 92% | 90% | 0.98 | 0.91 – 1.05 | 0.540 |
| Children say there was soap to wash their hands with (last time) | 65% | 94% | 1.52 | 1.18 – 1.95 | <0.001 |
| Children say there was a garbage bin nearby (last time) | 34% | 72% | 2.11 | 1.49 – 2.98 | <0.001 |
| Children say other children treat the toilet well | 79% | 89% | 1.10 | 0.98 – 1.23 | 0.099 |
| Children say they are happy to share with children of the opposite gender | 47% | 51% | 1.08 | 0.80 – 1.46 | 0.607 |

**Taken from SDG core questions for monitoring WinS
**Indicators in italics were not used in the current analysis – see analytic methods below.*
